# An immunogenic NSCLC microenvironment is associated with favorable survival in lung adenocarcinoma

**DOI:** 10.18632/oncotarget.26748

**Published:** 2019-03-05

**Authors:** Kevin B. Givechian, Chad Garner, Steve Benz, Bing Song, Shahrooz Rabizadeh, Patrick Soon-Shiong

**Affiliations:** ^1^ NantOmics LLC, Culver City, CA 90232, USA; ^2^ NantHealth, Inc. NantWorks, Culver City, CA 90232, USA; ^3^ NantBioscience, Inc. NantWorks, Culver City, CA 90232, USA

**Keywords:** NSCLC, lung adenocarcinoma, lung squamous cell carcinoma, tumor immunology, tumor microenvironment

## Abstract

The tumor microenvironment consists of an intricately organized system through which immune cells and cancer cells may communicate to regulate anti-tumor immunogenicity. To this end, non-small cell lung cancer (NSCLC) has been shown to activate a variety of immunological mechanisms, thereby broadening our understanding of lung cancer immunobiology. However, while recent work has highlighted the importance of NSCLC immunology and prognosis, studies have not yet examined the tumor microenvironment (TME) globally in regards to the survival outcomes between two major NSCLC subtypes: lung adenocarcinoma (LUAD) and lung squamous cell carcinoma (LUSC). In the present study, we identify an immunogenic tumor microenvironment state in NSCLC that is enriched for the lung adenocarcinoma subtype. By utilizing TME cell enrichment scores and RNA-seq expression data, we show that the inflamed TME is associated with favorable patient survival in lung adenocarcinoma, but this does not hold true for lung squamous cell carcinoma. Moreover, differentially regulated pathways between immune-inflamed and immune-excluded tumors within LUAD and LUSC were not subtype specific. Instead, immune-inflamed LUSC samples possessed elevated immune checkpoint marker expression when compared to those of the LUAD samples, thereby offering a putative explanation for our prognostic observations. These results shed light on the immunological prognostic effects within lung cancer and may encourage further TME exploration between these two subtypes as the landscape of NSCLC therapy progresses.

## INTRODUCTION

Lung cancer is the leading cause of cancer-related deaths [1], although non-small cell lung cancer (NSCLC) immunotherapy has fortunately emerged as a relatively promising area of research. In particular, immune checkpoint blockade has found a efficacious niche within NSCLC [2]. However, much work remains to elucidate lung tumor immunobiology and how alternative tumor microenvironments (TME) can affect patient survival across different NSCLC subtypes.

The TME has surfaced as a fascinating area of study across various tumor types [3]. In fact, broad categorizations have been proposed to stratify variable levels of immunogenicity. Immune-inflamed TMEs (‘hot’ tumors) express high levels of cytotoxic lymphocytes and immune activation markers (e.g., CD8, PRF1) [3–5]. Tumors with this TME subtype are often associated with a favorable prognosis [6]. Conversely, patients with immune-excluded TMEs (‘cold’ tumors) exhibit the opposite trend in survival [3, 6–8]. More specifically, interesting spatial organizations, such as compartmentalized or mixed TMEs, and their unique associations with survival have also been shown [9]. Together, recent work has encouraged further prognostic analysis of the TME to better understand the immune, stromal, and cancer signals that are integral to the clinical aftermath of this highly complex environment.

In NSCLC, several studies have advanced our understanding of TME [10–12]. However, despite being the two major subtypes, analysis of the TME within and between LUAD and LUSC remain scarce. Accordingly, the differential survival associations of immunogenicity within LUAD and LUSC have not yet been explored despite observations of differential immune activity between these subtypes [13–15]. In the present study, we sought to determine whether an immune-inflamed TME would be enriched for a certain NSCLC subtype and whether this would be reflected in patient overall survival. We find that immune-inflamed LUADs are associated with improved overall survival compared to their immune-excluded LUAD counterparts. However, this is not observed to be true for immune-inflamed LUSCs. Accordingly, we find that this profile of immune-inflamed profile of LUSC displays elevated immune checkpoint expression compared to immune-inflamed LUADs, thereby offering a putative explanation for our prognostic observations.

## RESULTS

### Identification of an immunogenic tumor microenvironment enriched for lung adenocarcinoma

**Figure 1:**
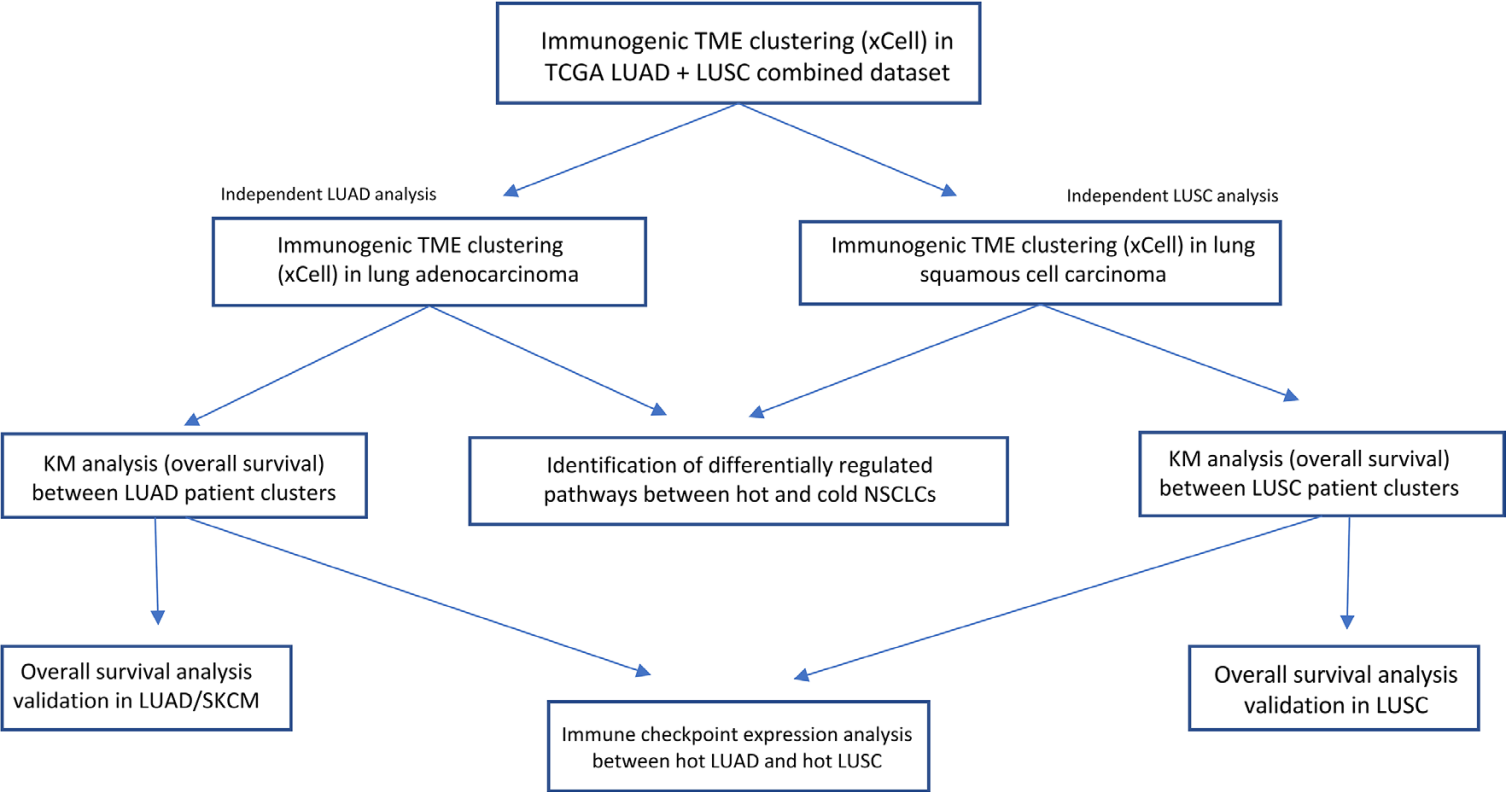
Experimental workflow of TME analysis in LUAD and LUSC patient tumor samples

**Figure 2:**
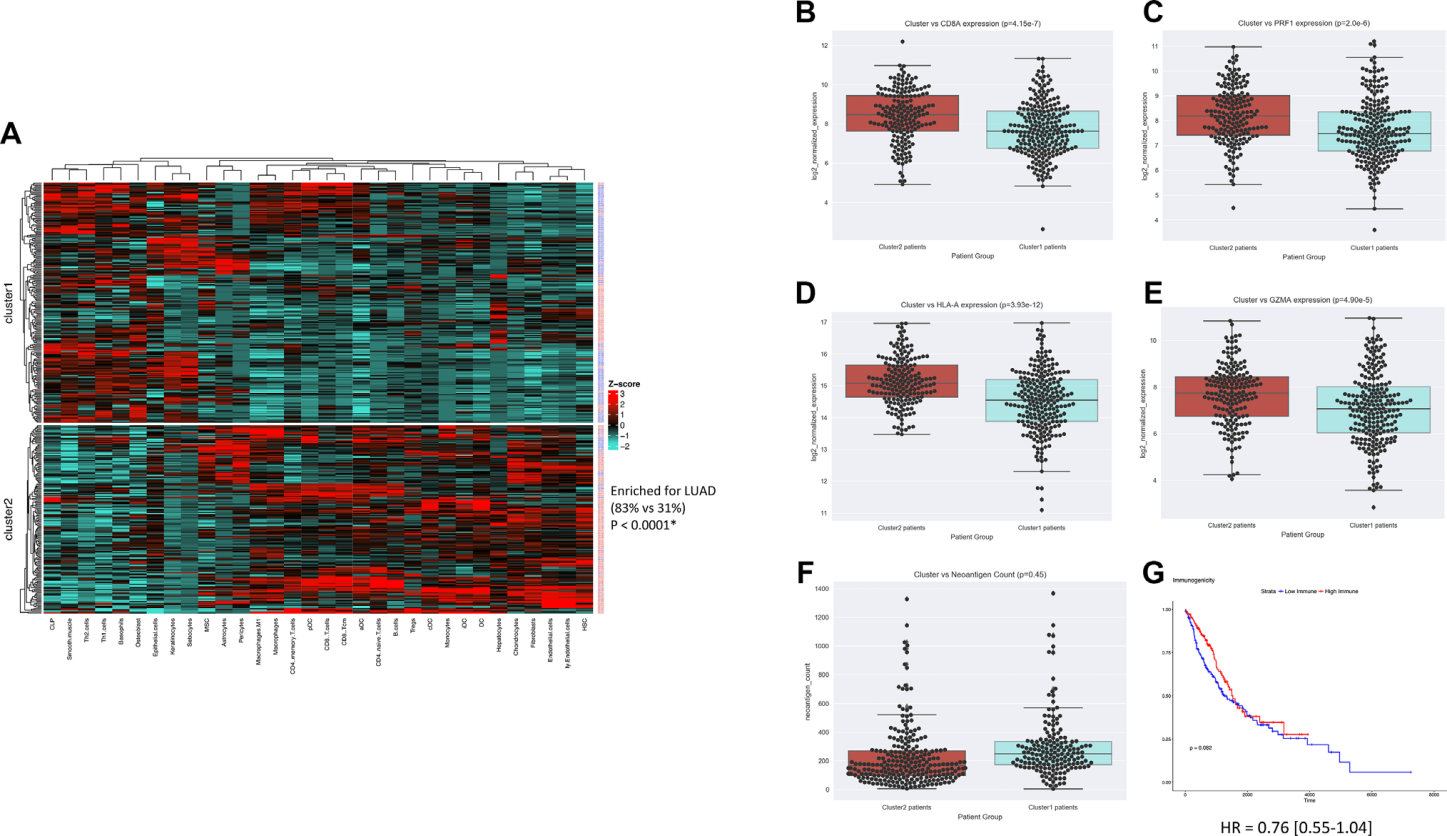
Combined LUAD and LUSC clustering yields an immunogenic cluster enriched for LUAD samples (**A**) Heatmap visualization of k-means clusters (*P* < 0.0001) (LUAD = red patient ID labels, LUSC= blue patient ID labels). K-means cluster vs immune-inflamed marker expression for (**B**) CD8A (*P* = 4.15e-7), (**C**) PRF1 (*P* = 2.0e-6), (**D**) HLA-A (3.93e-12), (**E**) GZMA (4.90e-5), and (**F**) neoantigen count (*P* = 0.45). (**G**) KM survival plot of cluster1 vs cluster2 (*P* = 0.08).

The overview of the study design is shown in Figure 1. In order to determine whether an immunogenic TME was enriched for LUAD vs LUSC tumor samples, unsupervised clusters were produced using immune and stromal cell type enrichment scores [16]. One cluster (cluster2) was significantly enriched for LUAD tumor samples (*P* < 0.0001, *x*^2^ = 113.9; Figure 2A). In order to determine whether the clusters produced were indeed indicative of immunogenic TMEs, we examined gene expression levels of several immunogenic activation markers between cluster1 and cluster2, namely CD8A, PRF1, HLA-A, and GZMA, which are shown to be favorably expressed in immunogenic tumors [4, 5, 17–21]. Expressions of these 4 genes were significantly elevated in cluster2 (*P* = 4.15e-7, *P* = 2.0e-6, 3.93e-12, 4.90e-5; Figure 2B–2E), which was enriched for LUAD. Neoantigen count was also compared between cluster1 and cluster2 since it is a molecular feature that is often associated with favorable immune phenotype [5, 22]. However, neither cluster was differentially associated with neoantigen count (*P* > 0.05; Figure 2F).

We next sought to explore whether there was a difference in survival between the two clusters produced. To do this, we first conducted overall survival (OS) analysis across LUAD and LUSC, to show that in the TCGA data used, neither lung cancer subtype was differentially associated with OS (*P* = 0.440, HR = 1.26 [0.84–1.52], Supplementary Figure 1A), thereby excluding subtype as a confounding variable. Overall survival between the two clusters did not differ, although the survival difference appeared to be trending towards significance (*P* = 0.082, HR = 0.76 [0.55–1.04]; Figure 2G). Considering that the immunogenic TME observed was enriched for LUAD samples, and that previous work has shown subtype dependent differences in LUAD and LUSC [13–15, 23, 24], we next sought to explore each subtype individually.

### An immunogenic TME is associated with favorable survival in LUAD but not in LUSC

**Figure 3:**
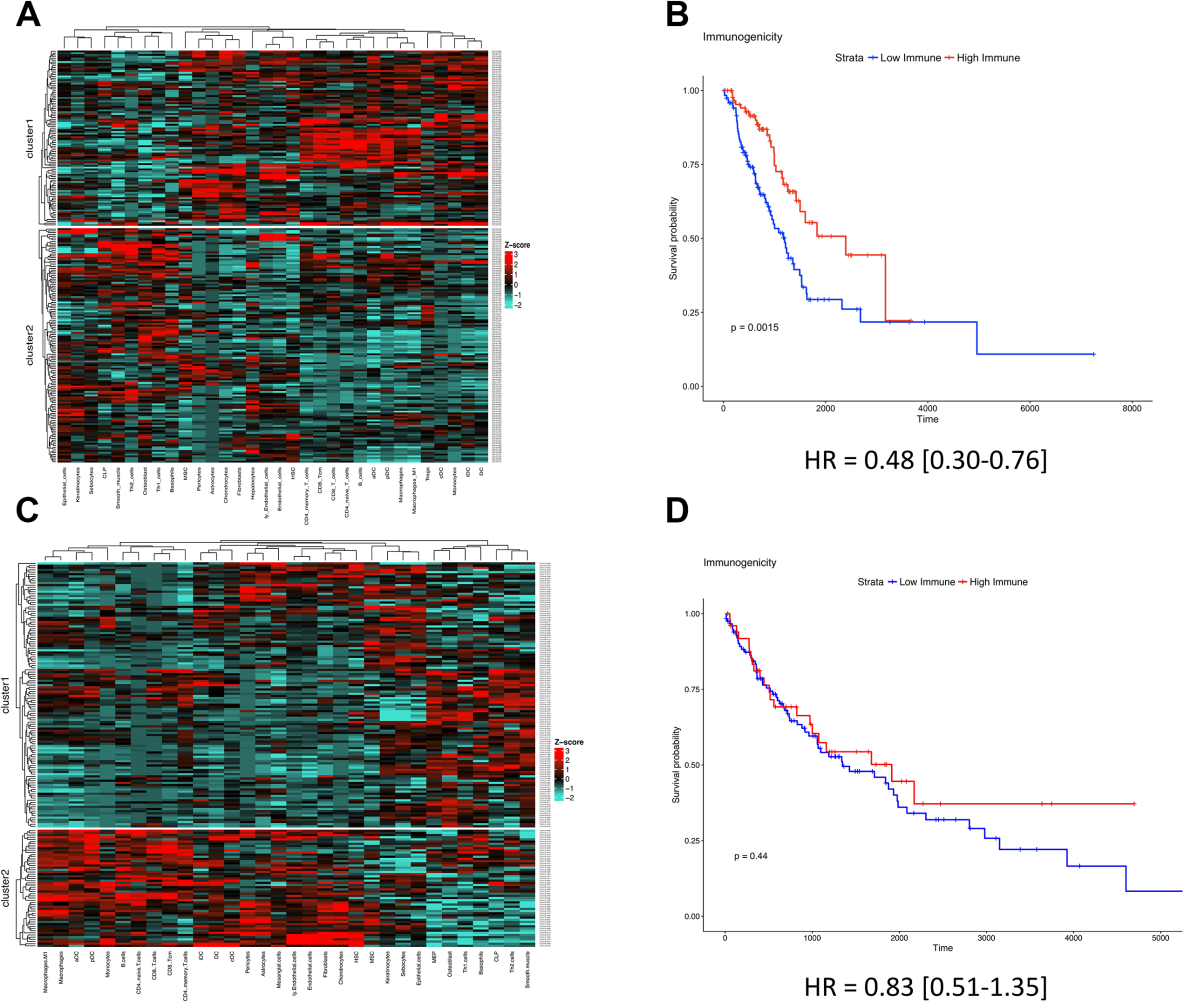
An immune-inflamed TME is associated with survival in LUAD but not in LUSC (**A**) LUAD clusters derived from cell type enrichment scores. (**B**) KM survival plot of LUAD cluster1 (red) vs LUAD cluster2 (blue) (*P* = 0.0018). (**C**) LUSC clusters derived from cell type enrichment scores. (**D**) KM survival plot of LUSC cluster1 (blue) vs LUSC cluster2 (red) (*P* = 0.44).

Unsupervised clusters were produced for both LUAD and LUSC independently. In LUAD, cluster1 possessed higher levels of immune cells known to help drive a favorable anti-tumor phenotype across various cancer types (e.g., CD8+ T-cells, M1-macrophages), indicative of a clinically favorable TME (Figure 3A) [20, 21, 25, 26]. This TME profile was also confirmed to possess higher levels of the cytolytic activation biomarkers CD8A, PRF1, HLA-A, and GZMA (*P* = 1.76e-10, *P* = 2.89e-10, *P* = 1.13e-5, and *P* = 1.01e-9; Supplementary Figure 1B–1E, respectively). Upon conducting OS analysis, we found that cluster1 patients (which possessed the relatively immunogenic TME) survived significantly longer than their less immunogenic counterparts (*P* = 0.0015, HR = 0.48 [0.30–0.76]; Figure 3B). These clusters were also shown to be unrelated to neoantigen count (*P* > 0.05, Supplementary Figure 1F).

When identical methods were applied within LUSC, cluster2 patients possessed higher levels of immune cells known to help drive a favorable anti-tumor effect (e.g., CD8+ T-cells, M1-macrophages; Figure 3C). This TME profile was also confirmed to possess higher levels of the cytolytic activation biomarkers CD8A, PRF1, HLA-A, and GZMA (*P* = 1.76e-10, *P* = 2.89e-10, *P* = 1.13e-5, and *P* = 1.01e-9; Supplementary Figure 2A–2D, respectively), and similarly, was not associated with neoantigen count (*P* > 0.05; Supplementary Figure 2E). However, interestingly, LUSC patients who possessed a relatively immunogenic TME did not survive significantly longer than their less immunogenic counterparts (*P* = 0.44, HR = 0.83 [0.51–1.35]; Figure 3D). These prognostic observations of LUAD and LUSC were also validated in a held-out LUAD data set (*P* = 0.02, HR = 0.60 [0.38–0.93]; Supplementary Figure 3A–3B), a held-out LUSC data set (*P* = 0.21, HR = 1.28 [0.87–1.87]; Supplementary Figure 3C–3D), and the TCGA melanoma data set (used as a positive control [27, 28]) (*P* = 0.0018, HR = 0.55 [0.38–0.80]; Supplementary Figure 3E–3F).

### Differentially expressed genes between hot and cold tumors are enriched for similar pathways in LUAD and LUSC

**Figure 4:**
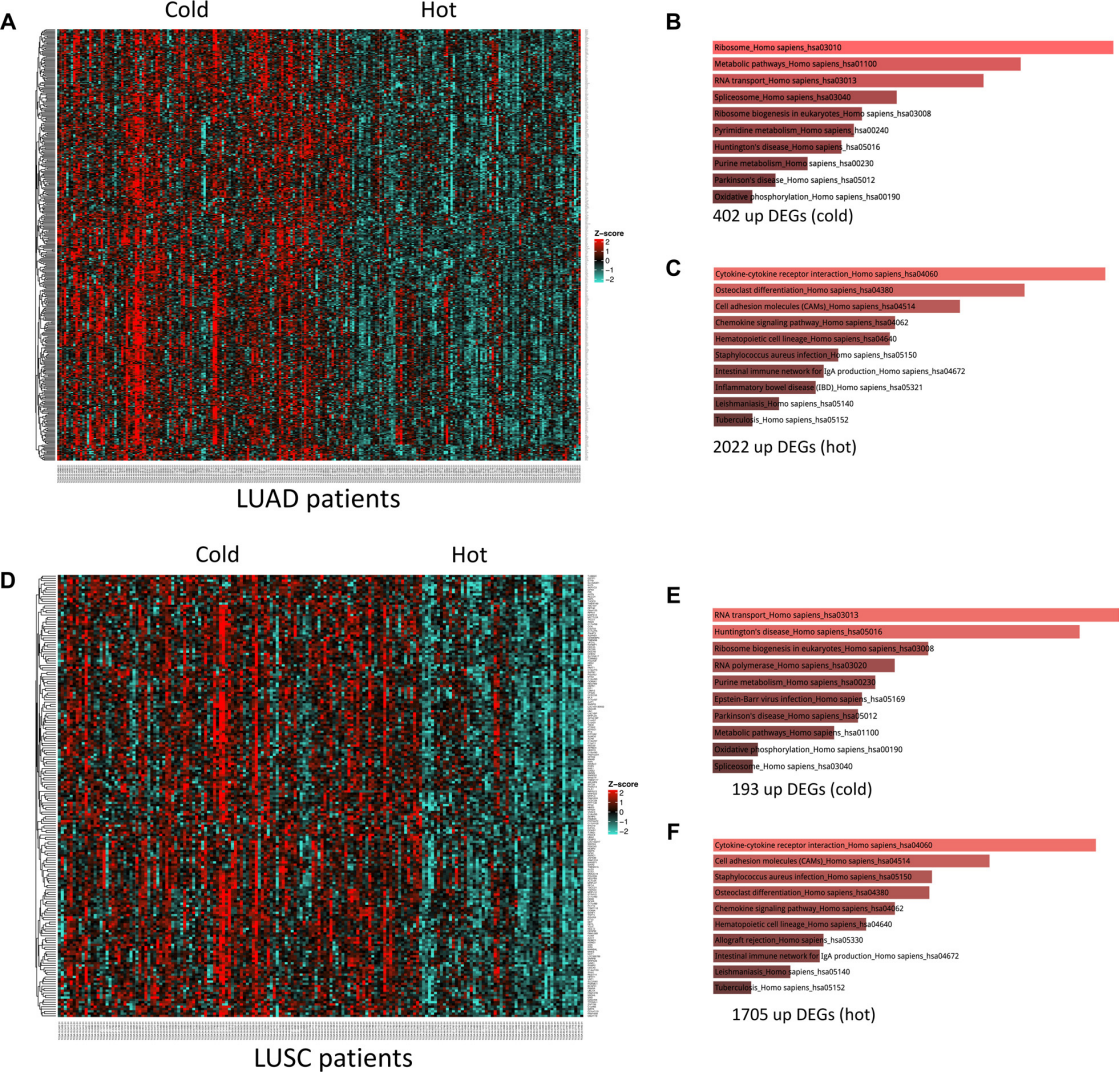
Differentially expressed genes (DEGs) and enriched pathways in hot vs cold tumor samples (**A**) heatmap of upregulated genes in cold vs hot LUADs. (**B**) enriched pathways of the 402 upregulated genes in cold vs hot LUADs. (**C**) enriched pathways of the 2022 upregulated genes in hot vs cold LUADs. (**D**) heatmap of upregulated genes in cold vs hot LUSCs. (**E**) enriched pathways of the 193 upregulated genes in cold vs hot LUSCs. (**F**) enriched pathways of the 1705 upregulated genes in hot vs cold LUSCs.

We then sought to determine whether the observed difference in survival could be attributed to activation of different pathways within each lung cancer subtype. In LUAD, 402 genes were significantly upregulated in the cold versus hot TMEs (Figure 4A). These genes were enriched for ribosomal and metabolic pathways, RNA transport, as well as nucleotide metabolism, which is in line with previous studies (Figure 4B; Supplementary Table 1) [21, 29–32]. In LUAD, 2022 genes were upregulated in hot versus cold tumors. As expected, these genes were enriched for immune-related pathways such as cytokine-cytokine receptor interaction and chemokine signaling (Figure 4C; Supplementary Table 2).

When these methods were applied to cold versus hot LUSC tumor samples, 102 genes were differentially upregulated in cold tumors (Figure 4D). These genes were also enriched for RNA transport, ribosomal, metabolic, and nucleotide metabolism pathways (Figure 4E; Supplementary Table 3). Furthermore, the 1705 upregulated genes in the hot LUSC tumors were enriched for similar pathways as observed in LUAD (Figure 4F; Supplementary Table 4). This suggested that the differential prognostic association observed was not due to upregulated pathways unique to LUAD or LUSC.

### Immunosuppressive checkpoint biomarker expression is elevated in hot LUSC tumors

**Figure 5:**
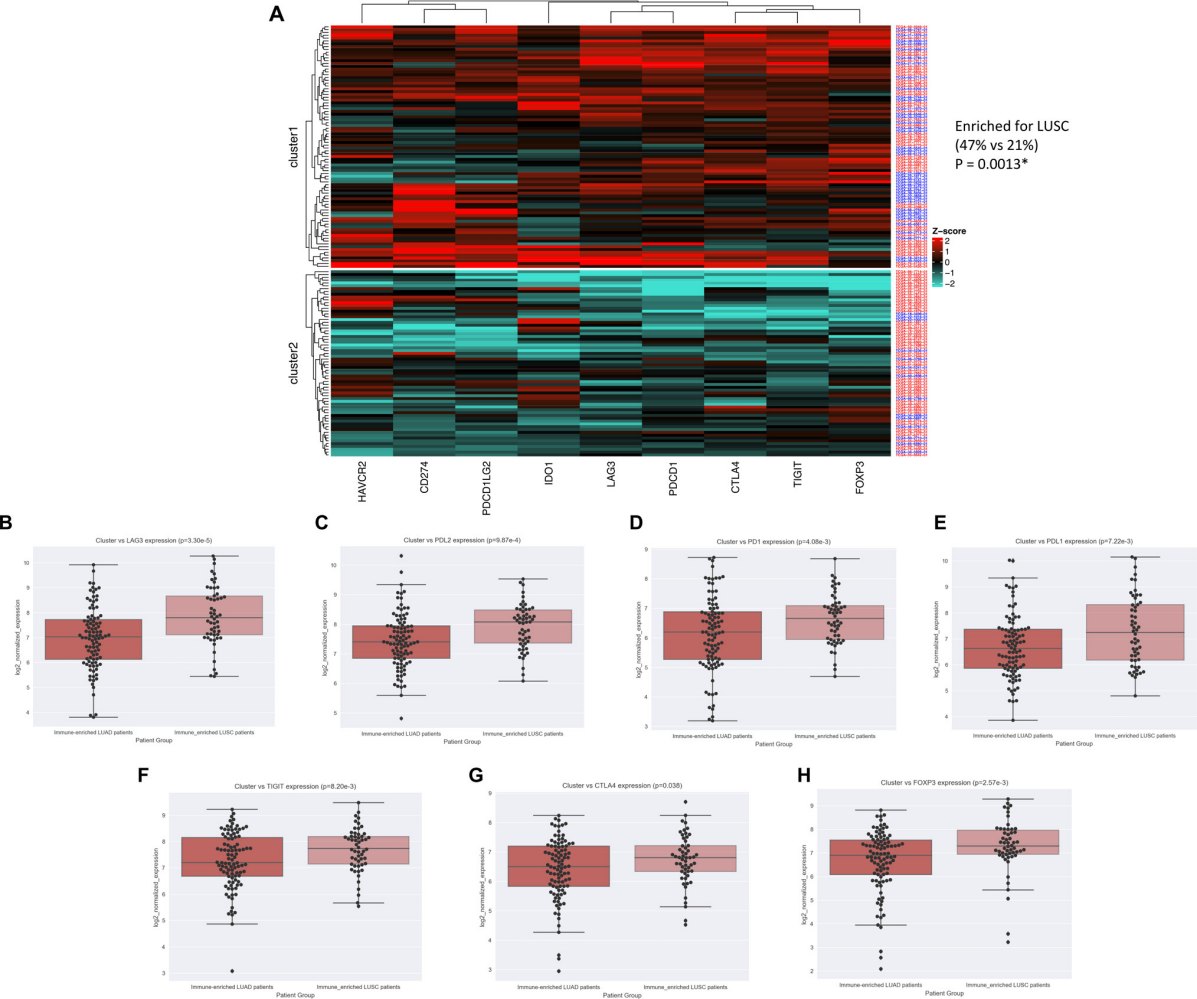
LUSC is enriched for immune checkpoint expression (**A**) Heatmap visualization of k-means clusters (*P* = 0.0013) (LUAD = red patient ID labels, LUSC= blue patient ID labels). NSCLC subtype vs immune-checkpoint marker expression for (**B**) LAG3 (*P* = 3.30e-5), (**C**) PDL2 (*P* = 9.87e-4), (**D**) PD1 (*P* = 4.08e-3), (**E**) PDL1 (*P* = 7.22e-3), (**F**) TIGIT (*P* = 8.20e-3), (**G**) CTLA4 (*P* = 0.038), and (**H**) FOXP3 (*P* = 2.57e-3).

In light of the pathway analysis results, we reasoned that perhaps the overall survival differences observed could be explained by differences in immunosuppression, so we examined expression differences of several well-characterized immune checkpoint genes [33–39]. To explore this in an unsupervised manner, we first combined hot LUSC with hot LUAD samples and produced k-means clusters using nine immune checkpoint gene expressions: PD1, PDL1, CTLA4, PDL2, LAG3, IDO1, TIGIT, TIM3, FOXP3. This yielded two distinct clusters, one of which (cluster1) was enriched for LUSC samples (*P* = 0.0019, *x*^*2*^ = 9.69; Figure 5A). Moreover, a direct comparison of these genes between hot LUSC and hot LUAD tumors revealed that 7 of 9 of these immune checkpoint genes (LAG3, PDL2, PD1, PDL1, TIGIT, CTLA4, FOXP3) were significantly elevated in LUSC tumor samples (*P* = 3.30e-5, *P* = 9.87e-4, *P* = 4.08e-3, *P* = 7.22e-3, *P* = 8.20e-3, *P* = 0.038, *P* = 2.57e-3; Figure 5B–5H, respectively).

## DISCUSSION

In the current study, we explored the prognostic associations of an immune-inflamed TME state across two main subtypes of NSCLC. As a result, we found that an immunogenic TME is uniquely associated with patient overall survival in LUAD as opposed to LUSC. An important aspect of our study is that we considered the holistic state of the TME rather than any one cell type and its prognostic association alone. This unsupervised approach used was able to cluster patients with high co-enrichment scores of the following cell types that have also been individually reported in association with favorable survival, such as M1 macrophages, activated dendritic cells, B cells, CD4+ T-cells, and CD8+ T-cells [12, 40–42]. This co-enrichment methodology also showed that markers of immune activation were consistently associated with the immune-inflamed cluster regardless of k-means features used or the subtype examined. Moreover, these methods may encourage further efforts to capture the TME state in regards to its overall co-enrichment landscape with immune and stromal cells as opposed to individual immune cells, which may be differentially associated with prognosis in alternative contexts [21, 43].

In regards to the differential pathway analysis, it is interesting to note the consistent association between the immune-excluded tumors, purine/pyrimidine metabolism, and metabolic activity. This is in line with recent work that highlights intratumoral metabolic crosstalk and how this communication may impair antitumor immunity [29, 44, 45]. Furthermore, we previously reported an association between less immunogenic tumors and nucleotide metabolism across five different tumor types [21], which is also consistent with our pathway analysis findings in the present study. Future work may include whether there exist targetable metabolic vulnerabilities that may result in enhanced immune infiltration into otherwise immune-excluded NSCLCs.

While examining the possible pro- or anti-tumor roles of each TME cell type is beyond the scope of our holistic TME study, we found it interesting that the 32-cell feature sets consistently included both Th1 and Th2 cells. These two helper T cell subtypes were co-enriched in the same patient clusters, despite being thought to be associated with different processes; that is, Th1 cells induce IFNG expression and anti-tumor effects while Th2 release IL-4 and IL-10, which help allow for tumor-escape [46, 47]. Whilst the less immunogenic and less favorably surviving LUAD patients showed elevated enrichment of both Th1 and Th2 cells, this result is in line with a previous study using different methods (e.g., flow cytometry) to show that high levels of Th1 in the TME is associated with worse prognosis in NSCLC [47]. Lastly, considering the relative scarcity of studies investigating the clinical differences between Th1 and Th2 cells in different TME contexts [48, 49], it is possible that this Th1-Th2 balance could mark an avenue worthy of further prognostic investigation, either alone or while considering potential tumor promoting metabolic pathways previously mentioned.

When examining immune checkpoint marker expression, the immune-inflamed LUSC tumors expressed significantly higher levels of seven genes known to inhibit the anti-tumor immune response. Of these seven, LAG3 was most significant, in line with previous work showing its increased expression in non-adenocarcinomas of NSCLC [50]. Moreover, LAG3 is known to be expressed on the surface of CD4+ and CD8+ cells, implicating the possibility that the tumor-infiltrating lymphocytes (TILs) within immune-inflamed LUSCs of our study were in a more exhausted state than immune-inflamed LUADs [38, 51]. This may thereby offer an explanation as to why immune-inflamed LUSCs were not associated with favorable prognosis. Furthermore, considering the growing body of evidence that patients with pre-existing immunity respond better to immunotherapy [52], our findings may encourage prospective validation in which LUSC patients with pre-existing immune-inflammation are selected for treatment with immunotherapy given their higher expression of ICB target genes.

There are several limitations to our study. First, the TME states used to cluster patient tumor samples were largely dependent on xCell output accuracy. While this tool has been shown to outperform other immune cell scoring methods, it is unable to infer the spatial TME architecture of a sample, which may differ between LUAD and LUSC. Second, our immune checkpoint marker analysis was limited to several genes that are currently well-characterized in regards to immunosuppression. However, this list of candidate genes may evolve as our understanding of cancer immunology progresses. Third, the data analyzed does not have matched treatment labels recorded for each patient. While data analyzed was pre-treatment transcriptomic/genomic data, it is possible that differential treatment-related effects on the TME, combined with treatment use for LUAD vs LUSC, could possibly influence our results. However, an important feature of our study to note is that our prognostic results are not confounded by immunotherapy use–the data analyzed was generated prior to the invention and standard clinical use of checkpoint inhibitor drugs, making ICB drug use an unlikely confounding factor. To this end, based on previous work [52], we speculate that OS significance levels would become more dramatic for LUAD patients if they with treated immune checkpoint inhibitor drugs since immune-inflamed tumors are suggested to present better candidates for immunotherapy.

In conclusion, our study highlights the prognostic impact of an immune-inflamed TME within LUAD as opposed to LUSC. We show that despite similar pathway expression, immune checkpoint marker expression seems to distinguish a LUSC subtype devoid of prognostic benefit in the context of immune-inflammation. This may encourage further exploration of potential treatable differences that may benefit one NSCLC subtype over the other. Furthermore, our unsupervised analysis of the TME in relation to survival may inspire functional validation of the pertinent immune cells that could be driving the prognostic trends observed.

## MATERIALS AND METHODS

### TME clustering

The cell-type deconvolution tool xCell has become an increasingly popular tool to infer cell-type enrichment scores from RNA expression data. In short, xCell is a computational tool that outputs enrichment scores for 64 immune and stromal cell types. It is a gene signature-based method that takes RNA-seq data as input and employs a curve fitting approach to linearly compare 64 immune and stromal cell types within a tumor sample. xCell introduces a novel spillover compensation method for separating these cell types and outperforms other methods in part by integrating the advantages of gene set enrichment and deconvolution approaches [16]. We chose to include both immune and stromal cells in the analysis as both types have been shown to influence the context of the tumor microenvironment [53]. However, as opposed to using all 64 cell types as input features, we only used the highly variable 32 cell types as our feature cell set (median standard deviation cutoff for the 64 immune enrichment scores within the dataset for the clusters produced). We only used the 32 highly variable cells because we hypothesized that the clinical variability (e.g., overall survival) would be strongly reflected by the enrichment variability of the TME cells themselves; we believed the cells with higher variability could better capture alternatively immunogenic tumor microenvironments.

K-means is a popular vector quantization method within the domain of machine learning that we used to produce unsupervised clusters (k = 2) using xCell enrichment scores as input features [16]. This was executed via the ComplexHeatmap package in R [54]. When LUAD and LUSC were combined, the highly variable cell types across the entire dataset (LUAD and LUSC combined) were used as input features to produce clusters. Proportional LUAD/LUSC enrichment significance was determined using the “N-1” Chi-squared test (*P* < 0.05 was considered significant, DF =1) [55, 56]. When each subtype was explored independently, the input features (highly variable cells) were obtained from within their respective subtype. However, to ensure that the results were not subtype-feature dependent, LUSC input features were validated in LUAD (Supplementary Figure 4A, 4B), and LUAD features were validated in LUAD (Supplementary Figure 4C–4D), showing that the observed prognostic results were independent of potential tissue-specific input features.

### Survival analysis

Data used for overall survival analysis (OS) was downloaded from OncoLnc (oncolnc.org) [57]. This data was parsed in Python and Kaplan-Meir plots were then produced in R. The coxph survival function in R (survival/survminer package in R) was used for significance analysis, hazard ratios, and 95% confidence intervals (*P* < 0.05 was considered significant).

### NSCLC genomic data (discovery and validation)

The RNA-seq data used to analyze gene expression differences between k-means clusters produced was log2(x+1) transformed RSEM normalized count data downloaded from xenabrowser (https://xenabrowser.net/datapages/, datasets: TCGA.LUAD.sampleMap/HiSeqV2 and TCGA.LUSC.sampleMap/HiSeqV2). For LUAD, there were 517 tumor samples with available RNA-seq data, and for LUSC, there were 501 tumor samples with available RNA-seq data (clinical data available at http://www.cbioportal.org/datasets). However, in the discovery datasets, only samples that also had available mutation data (and thus inferred neoantigen counts [58]) were included. This provided 230 LUAD samples and 176 LUSC samples with matched RNA-seq and neoantigen data (1 outlier LUSC sample was removed for visualization; results were not affected). Patient neoantigen data was downloaded from TCIA (neoantigen methods previously described [58]).

The held out LUAD and LUSC data sets consisted of patient tumor samples that did not have available mutation/neoantigen data which used earlier on. This left 287 LUAD samples and 324 LUSC samples that had available RNA-seq (and thus xCell scores) as validation sets. We performed this validation to show that the results we observed were not specific to the initial datasets examined and increase our overall sample size. Furthermore, to help ensure robustness of the clustering method we used, we applied these methods to the TCGA SKCM dataset as a positive control. We used SKCM because it is shown to also harbor prognostic associations between immunogenicity and patient survival [27, 28].

### Gene expression and pathway analysis

Patient RNA-seq expression values were accessed using the fbget API (https://confluence.broadinstitute.org/display/GDAC/fbget) and parsed using custom Python scripts. Two-tailed t-tested were used to assess differentially expressed genes between tumor samples of the different clusters (*P* < 0.05 was considered significant). The Seaborn and Matplotlib libraries were used to produce and visualize expression swarm-boxplots.

To identify upregulated genes in immune-inflamed and immune-excluded tumors, whole transcriptomic analysis was performed to surface the differentially expressed genes (acceptable *P* < 2.4e-6). These genes were then used as inputs in the Enrichr pathway analysis tool to identify enriched Kegg pathways in a given cluster (adjusted *P* < 0.05 was considered significant). Heatmaps were produced using the ComplexHeatmap package in R, and pathway bar plots were obtained using the Enrichr tool [59].

## SUPPLEMENTARY MATERIALS FIGURES AND TABLES



## Abbreviations

NSCLC: non-small cell lung cancer
LUAD: lung adenocarcinoma
LUSC: lung squamous cell carcinoma
OS: overall survival
TME: tumor microenvironment
