# Supplementary material for: An immunogenic NSCLC microenvironment is associated with favorable survival in lung adenocarcinoma
Source: Oncotarget. 2019 Mar 5;10(19):1840–9. doi: 10.18632/oncotarget.26748 (PMC6442995; doi:10.18632/oncotarget.26748)
Supplement: Supplementary file 1 [file oncotarget-10-1840-s001.pdf]

An immunogenic NSCLC microenvironment is associated with favorable survival in lung adenocarcinoma

SUPPLEMENTARY MATERIALS

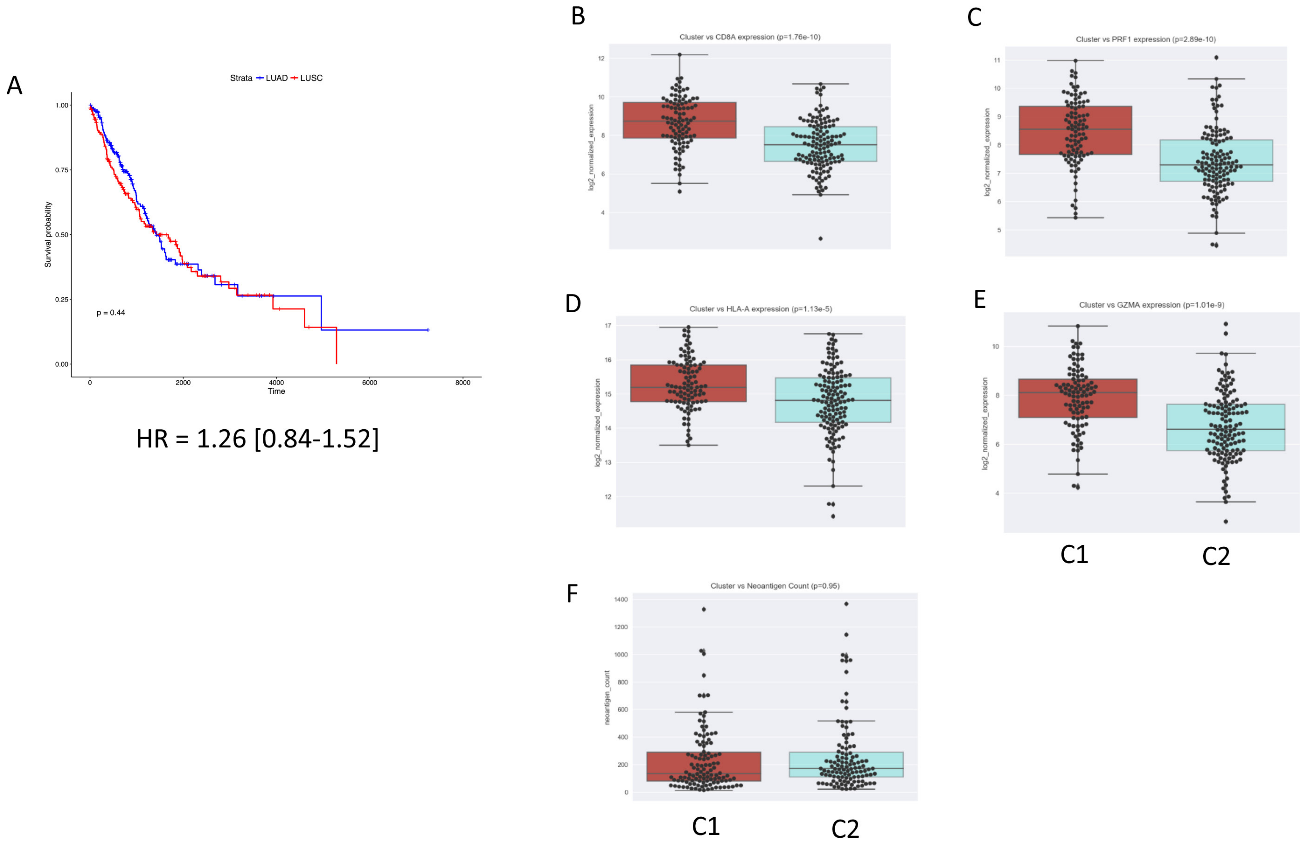

Supplementary Figure 1: Immune gene expression and neoantigen count analysis between unsupervised clusters produced from LUAD samples. Immune marker analysis between clusters produced within LUAD. First, we show that (**A**) neither lung cancer subtype was differentially associated with OS ($P = 0.440$, HR = 1.26 [0.84–1.52]. Gene expression levels of several immunogenic activation markers between cluster1 and cluster2: (**B**) CD8A, (**C**) PRF1, (**D**) HLA-A, and (**E**) GZMA were elevated in cluster1 LUAD tumors samples ($P = 1.76$e-10, $P = 2.89$e-10, $P = 1.13$e-5, and $P = 1.01$e-9, respectively). (**F**) Neither cluster is associated with neoantigen count ($P > 0.05$).

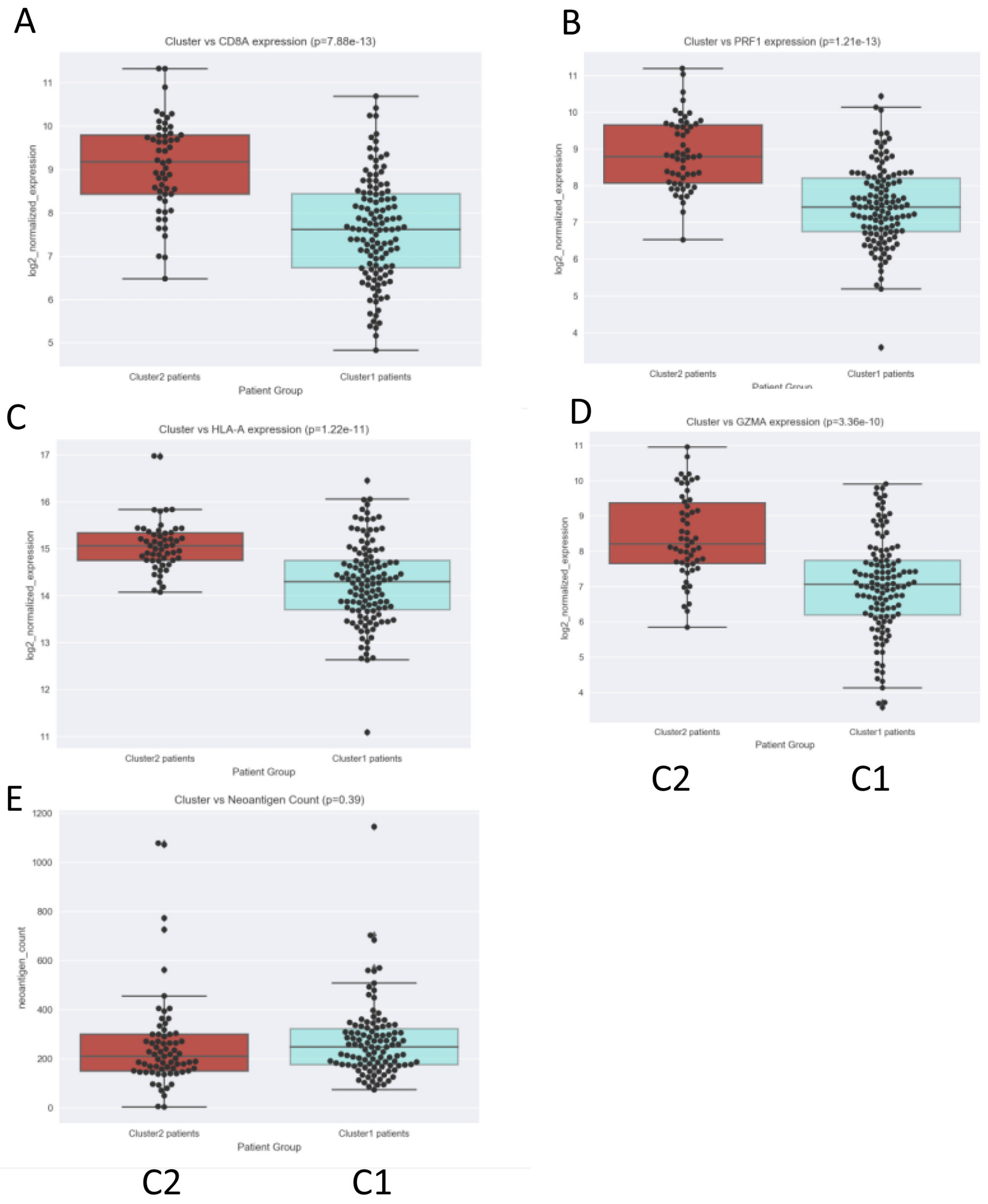

Supplementary Figure 2: Immune gene expression and neoantigen count analysis between unsupervised clusters produced from LUSC samples. Immune marker analysis between clusters produced within LUSC. Gene expression levels of several immunogenic activation markers between cluster1 and cluster2: (**A**) CD8A, (**B**) PRF1, (**C**) HLA-A, and (**D**) GZMA were elevated in cluster2 LUSC tumors samples (($P = 7.88e\text{-}13$ $P = 1.21e\text{-}13$, $P = 1.22e\text{-}11$, and $P = 3.36e\text{-}10$, respectively). (**E**) Neither cluster is associated with neoantigen count ($P > 0.05$).

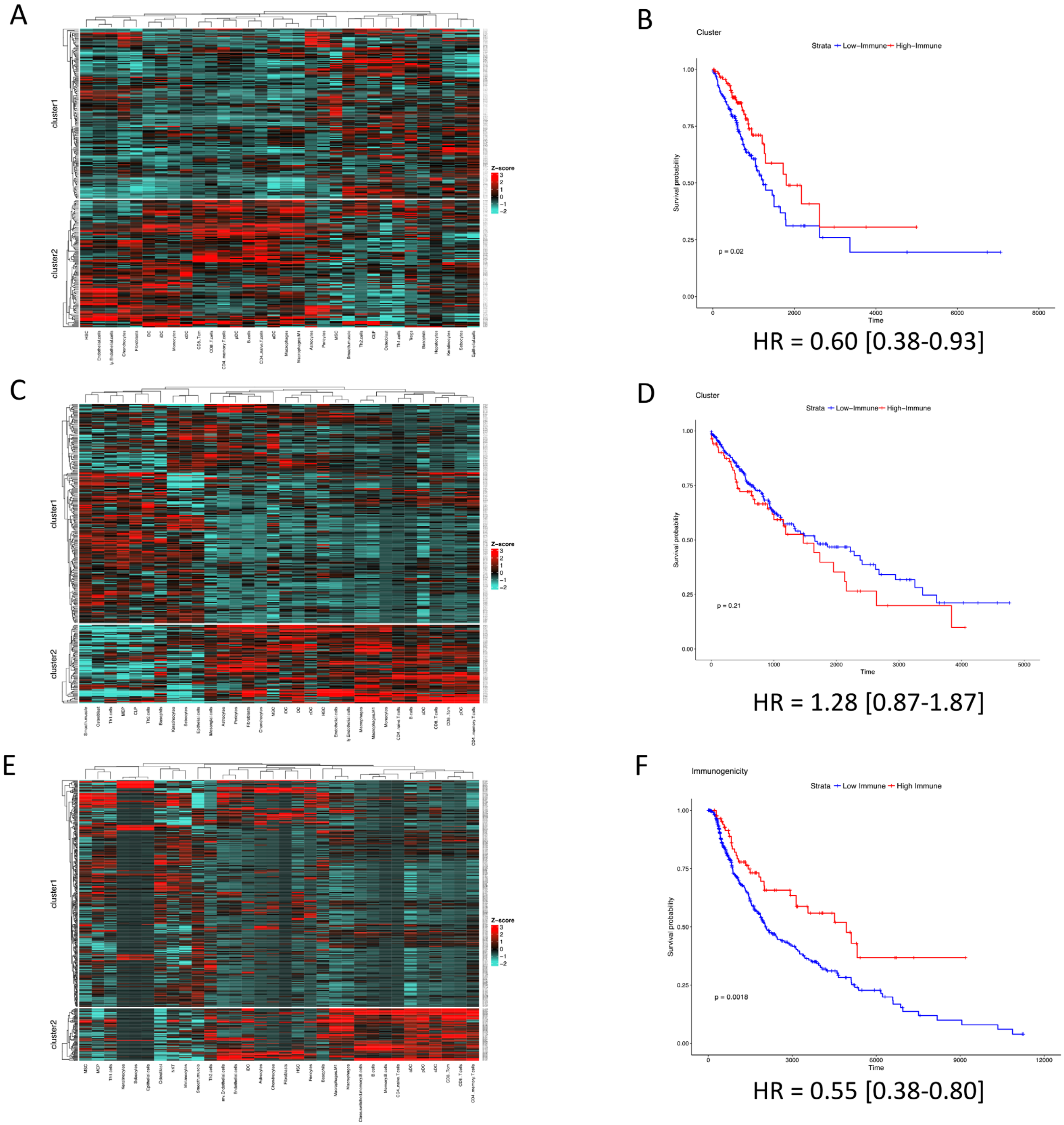

Supplementary Figure 3: Validation analysis using additional LUAD, LUSC, and melanoma samples. Overall survival validation analysis. Results from the study were again observed in (**A**, **B**) a held-out LUAD validation data set ($P = 0.02$, HR = 0.60 [0.38–0.93], (**C**, **D**) a held-out LUSC data set ($P = 0.21$, HR = 1.28 [0.87 – 1.87], and (**E**, **F**) the TCGA melanoma data set (used as a positive control [27, 28], $P = 0.0018$, HR = 0.55 [0.38–0.80]).

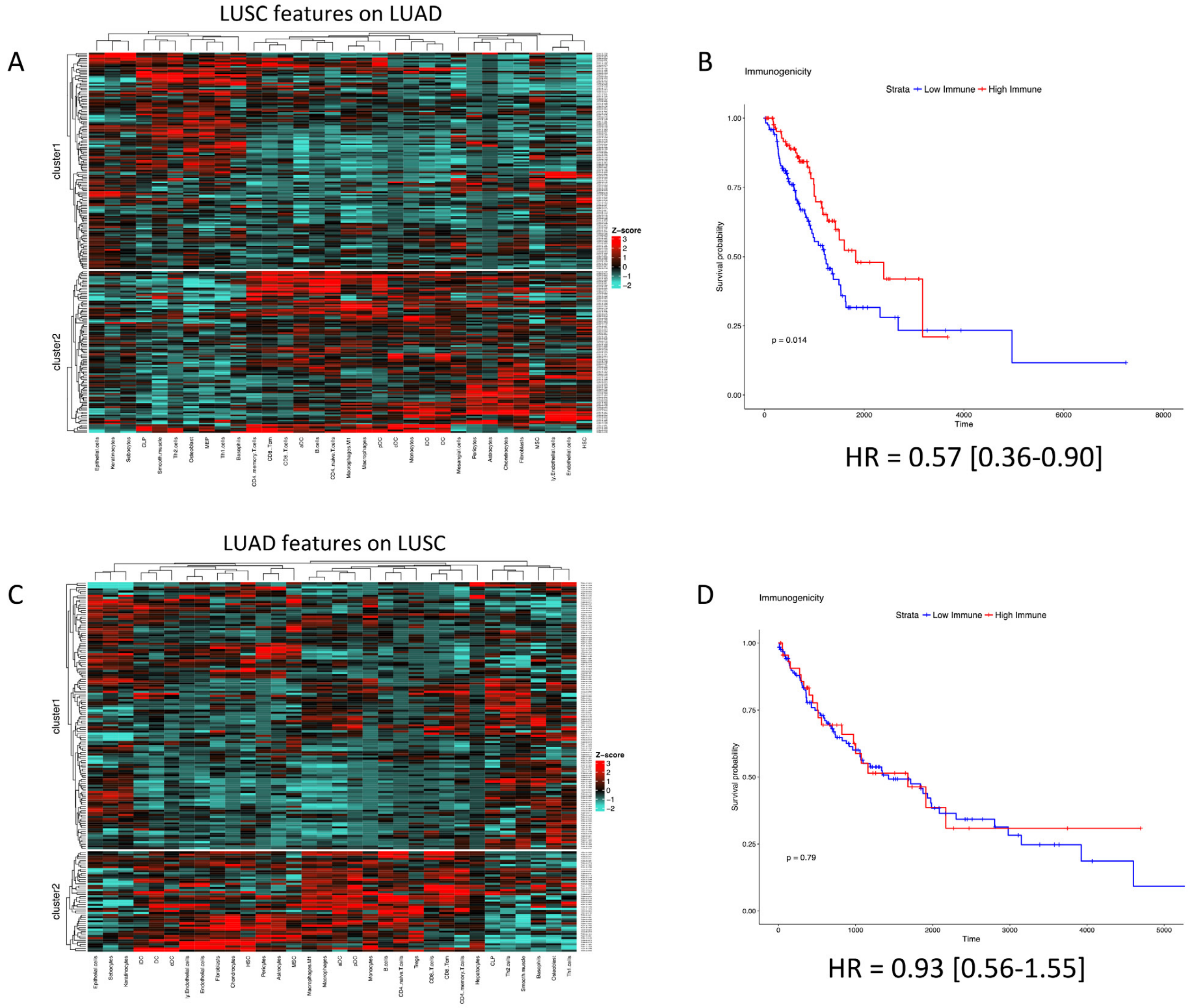

Supplementary Figure 4: TME-prognostic associations are not dependent on subtype-specific immune and stromal cell features. Immune and stromal feature independence. (**A**, **B**) LUSC input features were validated in LUAD ($P = 0.014$, HR = 0.57 [0.36–0.90]), and (**C**, **D**) LUAD features were validated in LUSC ($P > 0.05$, HR = 0.93 [0.56–1.55]).

Supplementary Table 1: Kegg pathway enrichment analysis in cold LUAD samples

Kegg pathway	Overlap	*P*-value	Adj. *P*-value	Z-score	Combined score	Genes
Ribosome Homo sapiens_ hsa03010	15/137	1.13666E-07	1.8755E-05	−1.746138049	27.92074335	MRPS17;MRPS16;MRPS11;RPLP0; MRPS18A;MRPS2;MRPL15;MRPL12;MRPL24;MRPL10;MRPS5;MRPL30;MRPL3;MRPL2; MRPS9
Metabolic pathways Homo sapiens_ hsa01100	48/1239	9.94831E-06	0.000573434	−1.98675506	22.88365916	STAT3;ACADVL;ECHS1;AHCY;ACY1;SHMT2; NDUFA10;ATP5G3;COX6A1;ATP5G2;COX5A;PTS; NT5C;ATP5B;ATIC;FLAD1;POLD2;AFMID;GCNT2; IDH3B;CKMT1B;CYC1;POLR2H; MPST;PDHX;ALG8;MDH2;PTGES2;AKR1C3;EPT1;SORD;ALG3;TALDO1;PAICS;MCAT;PRDX6;COASY;TRIT1;DAK;UGDH; GALE;IMPDH2;HMBS;POLR1C;NDUFS1;UMPS;PIG; ITPA
RNA transport Homo sapiens_ hsa03013	14/172	1.04261E-05	0.000573434	−1.818646653	20.86206217	EIF2B5;EIF2B4;POP5;EIF4A1;EIF2S2;AAAS;SNUPN;FXR2;EIF3J;RPP21;NUP88;GEMIN6;RAN; WIBG
Spliceosome Homo sapiens_ hsa03040	11/134	8.50186E-05	0.003507017	−1.722670085	16.14596768	PUF60;SNRPD1;PCBP1; PRPF40B;SNRPE;SNRPA1; SNRPC;TXNL4A;LSM4; SNRPA;SNRPB
Ribosome biogenesis in eukaryotes Homo sapiens_ hsa03008	Aug-89	0.000424553	0.011675198	−1.835750254	14.25363612	NOP56;POP5;NOP58; WDR75; MPHOSPH10;IMP4; RAN;GNL3
Pyrimidine metabolism Homo sapiens_ hsa00240	9/105	0.000268826	0.008871255	−1.680203199	13.81370073	NT5C;PNPT1;TXNRD2; ENTPD6; POLR1C;POLD2;POLR2H;UMPS; ITPA
Huntington's disease Homo sapiens_ hsa05016	12/193	0.000555192	0.013086658	−1.752185995	13.13473205	ATP5B;CREB3L4;NDUFA10; VDAC2;NDUFS1;CYC1;ATP5G3; POLR2H;COX6A1;ATP5G2; COX5A;SOD1

Pathway analysis results using Kegg gene sets from upregulated genes in cold LUAD samples when compared to hot LUAD samples.

Supplementary Table 2: Kegg pathway enrichment analysis in hot LUAD samples

Kegg pathway	Overlap	*P*-value	Adj. *P*-value	Z-score	Combined score
Cytokine-cytokine receptor interaction Homo sapiens_hsa04060	97/265	2.99958E-31	7.88891E-29	−1.877181663	131.9314515
Osteoclast differentiation Homo sapiens_hsa04380	63/132	2.42781E-28	3.19258E-26	−1.838268548	116.887025
Cell adhesion molecules (CAMs) Homo sapiens_hsa04514	65/142	7.10402E-28	6.22785E-26	−1.67764771	104.8726478
Chemokine signaling pathway Homo sapiens_hsa04062	69/187	8.09701E-23	4.25903E-21	−1.824413491	92.80419556
Hematopoietic cell lineage Homo sapiens_hsa04640	47/88	3.37912E-24	2.22177E-22	−1.699208644	91.83275861
Staphylococcus aureus infection Homo sapiens_hsa05150	36/56	1.14784E-22	5.03138E-21	−1.627646943	82.22707419
Intestinal immune network for IgA production Homo sapiens_hsa04672	33/48	2.6964E-22	1.01307E-20	−1.600975135	79.51235918
Inflammatory bowel disease (IBD) Homo sapiens_hsa05321	37/65	1.26484E-20	4.15815E-19	−1.702813886	78.01741498
Leishmaniasis Homo sapiens_hsa05140	39/73	2.55883E-20	6.72974E-19	−1.5783542	71.20295119
Tuberculosis Homo sapiens_hsa05152	62/178	3.63131E-19	8.68212E-18	−1.560661857	66.26495982

Pathway analysis results using Kegg gene sets from upregulated genes in hot LUAD samples when compared to cold LUAD samples.

Supplementary Table 3: Kegg pathway enrichment analysis in cold LUSC samples

Kegg pathway	Overlap	*P*-value	Adj. *P*-value	Z-score	Combined score	Genes
RNA transport Homo sapiens_ hsa03013	11/172	9.28028E-07	0.000104867	−1.867155195	25.93516614	POP5;NXT1;NCBP2;RPP40;SUMO2; RPP21;NMD3;THOC3;RAE1;RAN;EIF1
Huntington's disease Homo sapiens_ hsa05016	11/193	2.86693E-06	0.000161982	−1.876166193	23.94413662	COX8A;NDUFB9;NDUFB5;VDAC3; POLR2F;POLR2H;COX6A1;SLC25A5; POLR2I;ATP5G1;POLR2J
Ribosome biogenesis (eukaryotes) Homo sapiens_ hsa03008	6/89	0.000223416	0.006911083	−1.930702853	16.23040715	POP5;NXT1;EIF6;RPP40;NMD3;RAN
RNA polymerase Homo sapiens_ hsa03020	4/32	0.00024464	0.006911083	−1.746633355	14.52451813	POLR2F;POLR2H;POLR2I;POLR2J
Purine metabolism Homo sapiens_ hsa00230	8/176	0.000317897	0.007184474	−1.680606227	13.53523765	DGUOK;GMPS;POLR2F;HPRT1;POLR2H; POLR2I;POLR2J;HDDC3
Epstein-Barr virus infection Homo sapiens_ hsa05169	8/202	0.000790911	0.012767562	−1.799836314	12.85501634	PSMD14;SHFM1;POLR2F;POLR2H;POL R2I; POLR2J;RAN;MAPK13
Parkinson's disease Homo sapiens_ hsa05012	7/142	0.000464064	0.008739867	−1.648540408	12.65335315	COX8A;NDUFB9;NDUFB5;VDAC3; COX6A1;SLC25A5;ATP5G1
Metabolic pathways Homo sapiens_ hsa01100	23/1239	0.001979117	0.027955033	−1.837757742	11.44023362	COX8A;NDUFB9;PCYT2;AHCY;NDUFB 5;MMAB; GSS;DCXR;MPI;GMPS;AKR1C3;ALG3; COX6A1; ATP5G1;COASY;PTS;DGUOK;NSDHL;P OLR2F; HPRT1;POLR2H;POLR2I;POLR2J

Pathway analysis results using Kegg gene sets from upregulated genes in cold LUSC samples when compared to hot LUSC samples.

Supplementary Table 4: Kegg pathway enrichment analysis in hot LUSC samples

Kegg pathway	Overlap	*P*-value	Adj. *P*-value	Z−score	Combined Score
Cytokine-cytokine receptor interaction Homo sapiens_hsa04060	91/265	1.33723E-32	3.59714E-30	−1.877181663	137.7703492
Cell adhesion molecules (CAMs) Homo sapiens_hsa04514	63/142	2.82108E-30	3.79435E-28	−1.701720853	115.785825
Staphylococcus aureus infection Homo sapiens_hsa05150	37/56	1.61149E-26	1.44497E-24	−1.749095362	103.8788646
Osteoclast differentiation Homo sapiens_hsa04380	56/132	7.56019E-26	5.08423E-24	−1.786504382	103.3391241
Chemokine signaling pathway Homo sapiens_hsa04062	65/187	5.82264E-24	2.29729E-22	−1.798475135	96.21893884
Hematopoietic cell lineage Homo sapiens_hsa04640	44/88	3.34955E-24	1.80206E-22	−1.669973041	90.26741412
Allograft rejection Homo sapiens_hsa05330	29/38	5.97807E-24	2.29729E-22	−1.523332272	81.45858483
Intestinal immune network for IgA production Homo sapiens_hsa04672	32/48	2.69366E-23	9.05744E-22	−1.552476653	80.67996888
Leishmaniasis Homo sapiens_hsa05140	37/73	7.83495E-21	2.34178E-19	−1.611535969	74.6071738
Tuberculosis Homo sapiens_hsa05152	57/178	3.13213E-19	7.65949E-18	−1.560661857	66.49574843

Pathway analysis results using Kegg gene sets from upregulated genes in hot LUSC samples when compared to cold LUSC samples.
